# Supplementary material for: Characterization of the Peroxisomal Proteome and Redox Balance in Human Prostate Cancer Cell Lines
Source: Antioxidants (Basel). 2024 Nov 1;13(11):1340. doi: 10.3390/antiox13111340 (PMC11591464; doi:10.3390/antiox13111340)
Supplement: Supplementary file 1 [file antioxidants-13-01340-s001.zip › Hussein et al_Supplementary Figure Captions.pdf]

antioxidants

MDPI

Article

Characterization of the Peroxisomal Proteome and Redox Balance in Human Prostate Cancer Cell Lines

Mohamed A. F. Hussein [1,2], Celien Lismont [1], Cláudio F. Costa [1], Hongli Li [1], Frank Claessens [3] and Marc Fransen [1,*]

[1] Laboratory of Peroxisome Biology and Intracellular Communication, Department of Cellular and Molecular Medicine, KU Leuven, 3000 Leuven, Belgium; mohamed.hussein@kuleuven.be (M.A.F.H.); celien.lismont@kuleuven.be (C.L.); claudiofcosta@live.com.pt (C.F.C.); hongli.li@kuleuven.be (H.L.)
[2] Department of Biochemistry, Faculty of Pharmacy, Assiut University, Asyut 71515, Egypt
[3] Laboratory of Molecular Endocrinology, Department of Cellular and Molecular Medicine, KU Leuven, 3000 Leuven, Belgium; frank.claessens@kuleuven.be
* Correspondence: marc.fransen@kuleuven.be; Tel.: +32-16-330114

Supplementary Caption

Figure S1. Representative images showing the distribution patterns of compartment-specific biosensors for monitoring GSSG/GSH and H_2O_2 levels. RWPE-1, 22Rv1, LNCaP, and PC3 cells were electroporated with a plasmid encoding peroxisomal (po-), mitochondrial (mt-), or cytosolic (c-) **(A)** roGFP2 or **(B)** roGFP2-Orp1. Cells were cultured in MEM and, in the case of RWPE-1, also in KSFM. Images were captured one to two days later. Scale bar, 10 µm.

Figure S2. Validation of a selected set of peroxisome-related proteomics data through immunoblotting. The relative abundances of 3-ketoacyl-CoA thiolase 1 (ACAA1), alpha-methylacyl-CoA racemase (AMACR), peroxin 13 (PEX13), and peroxin 14 (PEX14) among different cell lines were determined by mass spectrometry (MS) and immunoblotting (IB). Representative blots are shown, with relevant molecular mass markers displayed on the right. LCF serves as a protein loading correction factor for individual lanes, determined through membrane staining with Ponceau S. The values represent the mean ± standard deviation of 3 biological replicates, normalized relative to the RWPE-1/MEM condition. *, $p < 0.05$; **, $p < 0.01$; ***, $p < 0.001$; ****, $p < 0.0001$; ns, non-significant.

Figure S3. Validation of a selected set of antioxidant enzyme-related proteomics data through immunoblotting. The relative abundances of glutathione reductase (GSR), glutathione S-transferase P (GSTP1), peroxiredoxin 2 (PRDX2), and thioredoxin (TXN) among different cell lines were determined by MS and IB. Representative blots are shown, with relevant molecular mass markers displayed on the right. LCF serves as a protein loading correction factor for individual lanes, determined through membrane staining with Ponceau S. The values represent the mean ± standard deviation of 3 biological replicates, normalized relative to the RWPE-1/MEM condition. *, $p < 0.05$; **, $p < 0.01$; ***, $p < 0.001$; ****, $p < 0.0001$; ns, non-significant.

Figure S4. Comparative analysis of catalase localization between non-malignant and malignant prostate cell lines. RWPE1, 22Rv1, LNCaP, and PC3 cells were cultured in MEM or, in the case of RWPE-1, also in KSFM. Upon reaching 70-80% confluency, the cells were processed for immunofluorescence microscopy or harvested, lysed, and subjected to differential centrifugation to prepare light mitochondrial (L) and cytosolic (S) fractions. **(A)** Representative images depicting the distribution patterns of PEX14 and CAT. Nuclei were counterstained with DAPI. Scale bar, 10 µm. **(B)** Immunoblot analysis of the L- and S-fractions with antisera specific for the indicated proteins. Equal amounts of proteins were loaded. Specific protein bands and degradation products are indicated by arrows and arrowheads, respectively. **(C)** Densitometry quantifications of the relative normalized amounts of the proteins in the L-and S-fractions. The fractional distribution represents the average value ± standard deviation of 3 independent experiments. Statistical analysis was performed using two-way ANOVA but no significant differences were observed (ns, non-significant).

Figure S5. Evaluation of LNCaP cell viability following CAT inhibition or CAT expression knockdown using the sulforhodamine B assay. (A) LNCaP cell viability was assessed after treatment with 10 mM 3-AT, control DsiRNA (DsiNC), or CAT-targeting DsiRNA (DsiCAT1) using the

sulforhodamine B assay, with optical density measured at 510 nm ($n \geq 4$ technical replicates). Values were normalized to the baseline measurement (6 h post-seeding). Data are presented as mean values, with error bars representing standard deviation. **(B,C)** Validation of CAT inhibition by 3-AT treatment or CAT knockdown by DsiCAT1, respectively. Equal protein loading was confirmed using Ponceau S staining. Statistical significance was determined by an unpaired t-test (*, $p < 0.05$; **, $p < 0.01$; ***, $p < 0.001$).

Figure S6. Concentration-dependent effect of catalase knockdown on 22Rv1 cell proliferation. (A) Proliferation profiles of 22Rv1 cells treated with 50, 100, 150, and 200 nM DsiRNA targeting catalase. Each data point represents the mean fold change in confluence relative to the starting point ($n = 3$ technical replicates). Error bars represent standard deviation. Statistical analysis was assessed at each time point using the unpaired t-test (*, $p < 0.05$; **, $p < 0.01$). **(B)** Validation of CAT siRNA knockdown by immunoblotting. Ponceau S was used as a loading control.

Figure S7. Concentration-dependent effect of R1881 on LNCaP cell proliferation. LNCaP cells were treated with either a vehicle (ethanol) or varying concentrations of R1881. Each data point represents the mean fold change in confluence relative to the initial time point ($n= 3$ technical replicates). Error bars indicate standard deviation. Statistical significance was assessed at each time point using one-way ANOVA test (*, $p < 0.05$; **, $p < 0.01$; ****, $p < 0.0001$).

Figure S8. Representative images showing the distribution patterns of compartment-specific biosensors used to monitor GSSG/GSH and H_2O_2 levels following R1881 treatment in LNCaP cells. LNCaP cells were cultured in phenol red-free MEM supplemented with 10% (v/v) charcoal-stripped serum for 4 days. Subsequently, they were treated with either the ethanol vehicle (-) or 1 pM or 10 nM R1881 (+). After one day, the cells were transfected with a plasmid encoding a peroxisomal (po-), cytosolic (c-), or mitochondrial (mt-) variant of **(A)** the glutathione redox sensor roGFP2, or **(B)** the H_2O_2 sensor roGFP2-Orp1. Images were captured one to two days later. Scale bar, 10 μm.

Figure S9. Enzalutamide does not influence catalase activity or H_2O_2 levels in LNCaP cells cultured in a standard medium. LNCaP cells were treated with either DMSO (control; -) or 20 μM enzalutamide (+). After two days, the cells were transfected or not with a plasmid encoding a peroxisomal (PO), cytosolic (C), or mitochondrial (MT) variant of the H_2O_2 sensor roGFP2-Orp1. Subsequently, the medium was replaced with fresh DMSO- or enzalutamide-containing medium. Two days later, the cells were processed for analysis. **(A)** CAT activity ($n = 4$). Error bars represent the standard deviation, and statistical significance was assessed using an unpaired t-test (ns, non-significant). **(B)** F400/F480 response ratios of roGFP2-Orp1 are presented as box plots, with the values expressed as a percentage of the average vehicle response. Each box represents the interquartile range, with the bottom and top edges indicating the 25th and 75th percentile, respectively. The line inside the box represents the median, and the lines extending from the box show one standard deviation above and below the mean. Data from each independent experiment ($n = 3$) are color-coded. Statistical comparisons were performed using nested t-tests (ns, non-significant). AU, arbitrary units.

Figure S10. Impact of androgen receptor activity on the mRNA expression levels of CAT, ACOX1, and ACOX3 in AR-positive cell lines. Gene expression data were obtained from the Gene Expression Omnibus (GEO) database (www.ncbi.nlm.nih.gov/geo/) to evaluate the mRNA levels of catalase, ACOX1, and ACOX3 following treatment with R1181, dihydrotestosterone (DHT), and/or enzalutamide in LNCaP, VCaP, and/or 22Rv1 cells. Statistical analysis was performed using an unpaired t-test by GraphPad Prism 10.3.1., with only significant differences shown (*, $p < 0.05$; **, $p < 0.01$; ***, $p < 0.001$; ****, $p < 0.0001$).

Figure S11. Impact of CAT knockdown on AR expression and activity. LNCaP cells were treated with control DsiRNA (DsiNC) or CAT-targeting DsiRNA (DsiCAT1/2). Four days post-treatment, the cells were harvested for processing. **(A)** Immunoblot analysis of CAT, AR, and prostate-specific antigen (PSA), with molecular mass markers displayed on the right of each blot. Ponceau S was used as a loading control. **(B)** Densitometric quantification of protein bands from panel A. Data represent the mean ± standard deviation of 3 technical replicates. Statistical significance was determined using a one-way ANOVA test (ns, non-significant; ****, $p < 0.0001$).

Figure S12. Catalase expression in different prostate cancer tissue types. mRNA expression data were obtained from the University of Alabama at Birmingham Cancer Data Analysis Portal (UALCAN; https://ualcan.path.uab.edu/) to assess catalase levels in **(A)** primary disease, **(B)** various Gleason scores, **(C)** metastatic stages, and **(D)** specific molecular signatures. Statistical analysis from this database was also included (*, $p < 0.05$; **, $p < 0.01$; ***, $p < 0.001$; ****, $p < 0.0001$). ERG, ETS-related gene protein; ETV1/4, ETS variant transcription factor 1/4; FLI1, friend leukemia integration 1 transcription factor; FOXA1, forkhead box protein A1; IDH1, isocitrate dehydrogenase 1; N0, no regional lymph node metastasis; N1, metastases in 1 to 3 axillary lymph nodes; SPOP, speckle-type POZ protein; TPM, transcripts per million.

Figure S13. AR activation by R1881 reduces CAT protein levels in 22Rv1 cells. Cells were treated with ethanol (vehicle, -) or 10 nM R1881 (+). After 3 days, the cells were harvested and subjected to immunoblotting using antisera against catalase (CAT). **(A)** Representative blot, with relevant molecular mass markers indicated on the right. Ponceau S staining was used as a loading control. **(B)** Quantification of the data shown in panel A. Values represent the mean ± standard deviation of at least 3 technical replicates, normalized to the vehicle condition (-). Statistical significance was assessed using an unpaired t-test. ***, $p < 0.001$.
