# Supplementary figures and images for: Characterization of the Peroxisomal Proteome and Redox Balance in Human Prostate Cancer Cell Lines
Source: Antioxidants (Basel). 2024 Nov 1;13(11):1340. doi: 10.3390/antiox13111340 (PMC11591464; doi:10.3390/antiox13111340)

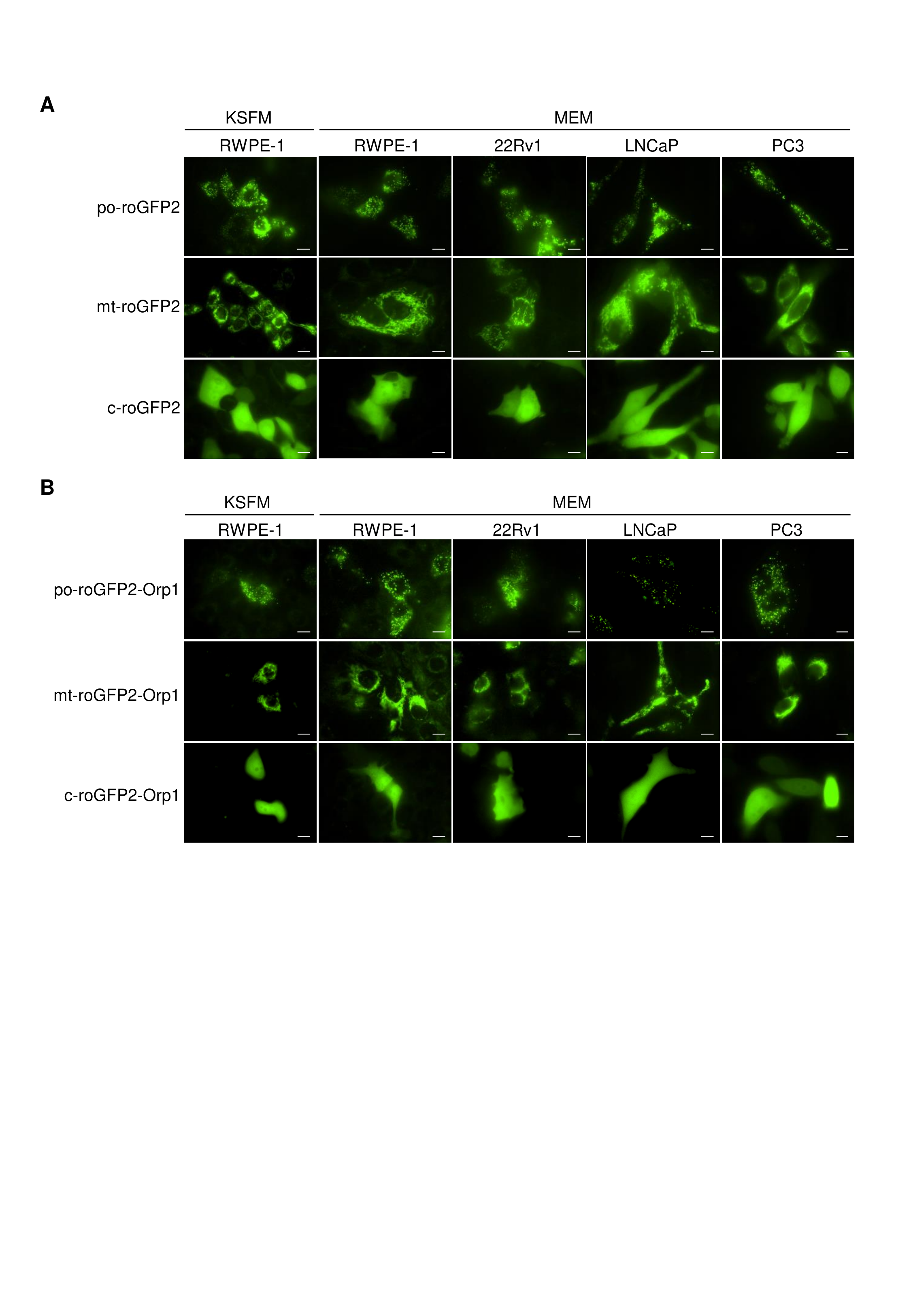

Supplement: Supplementary file 1 [file antioxidants-13-01340-s001.zip › Hussein et al_Figure S1.png]

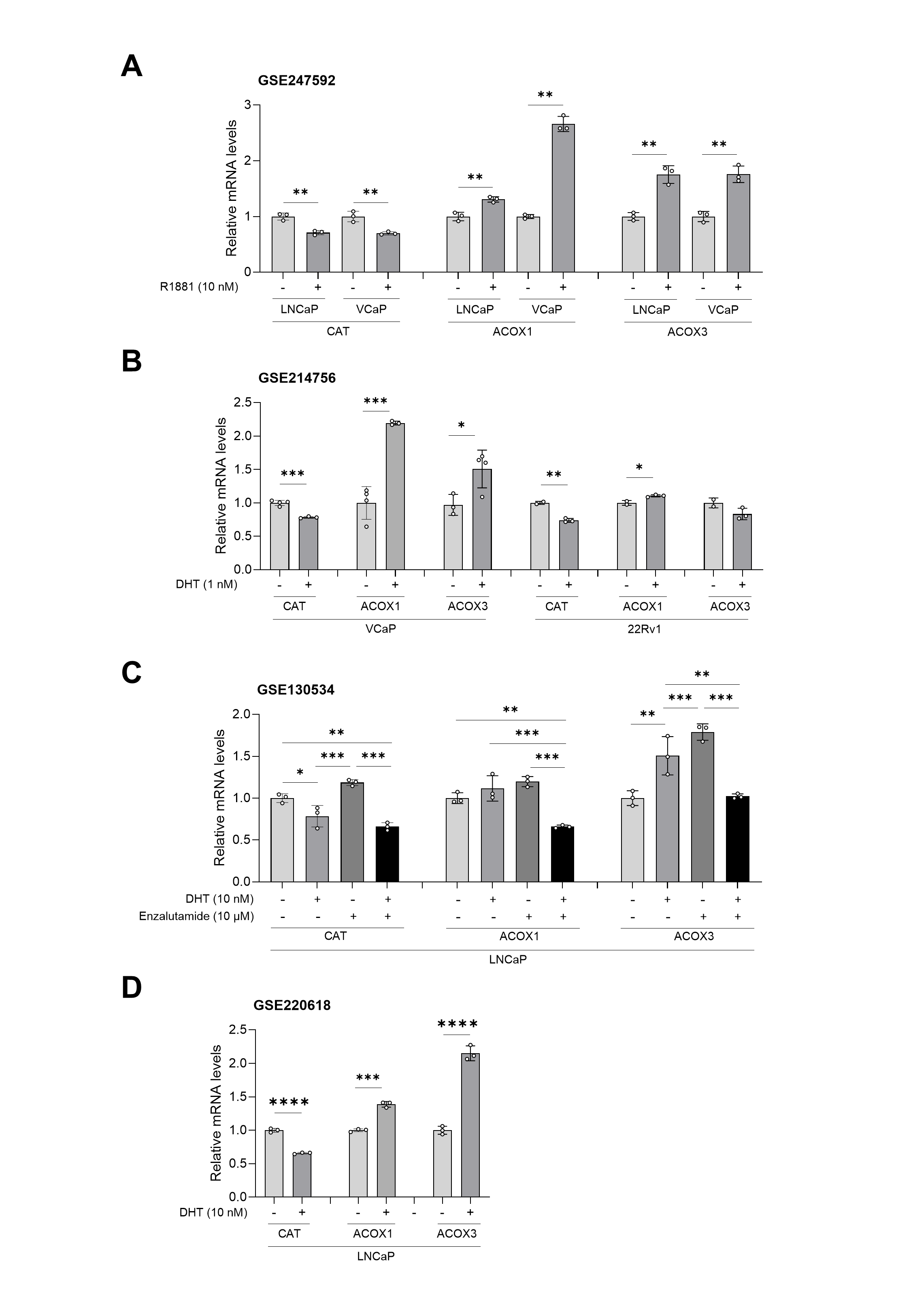

Supplement: Supplementary file 1 [file antioxidants-13-01340-s001.zip › Hussein et al_Figure S10.png]

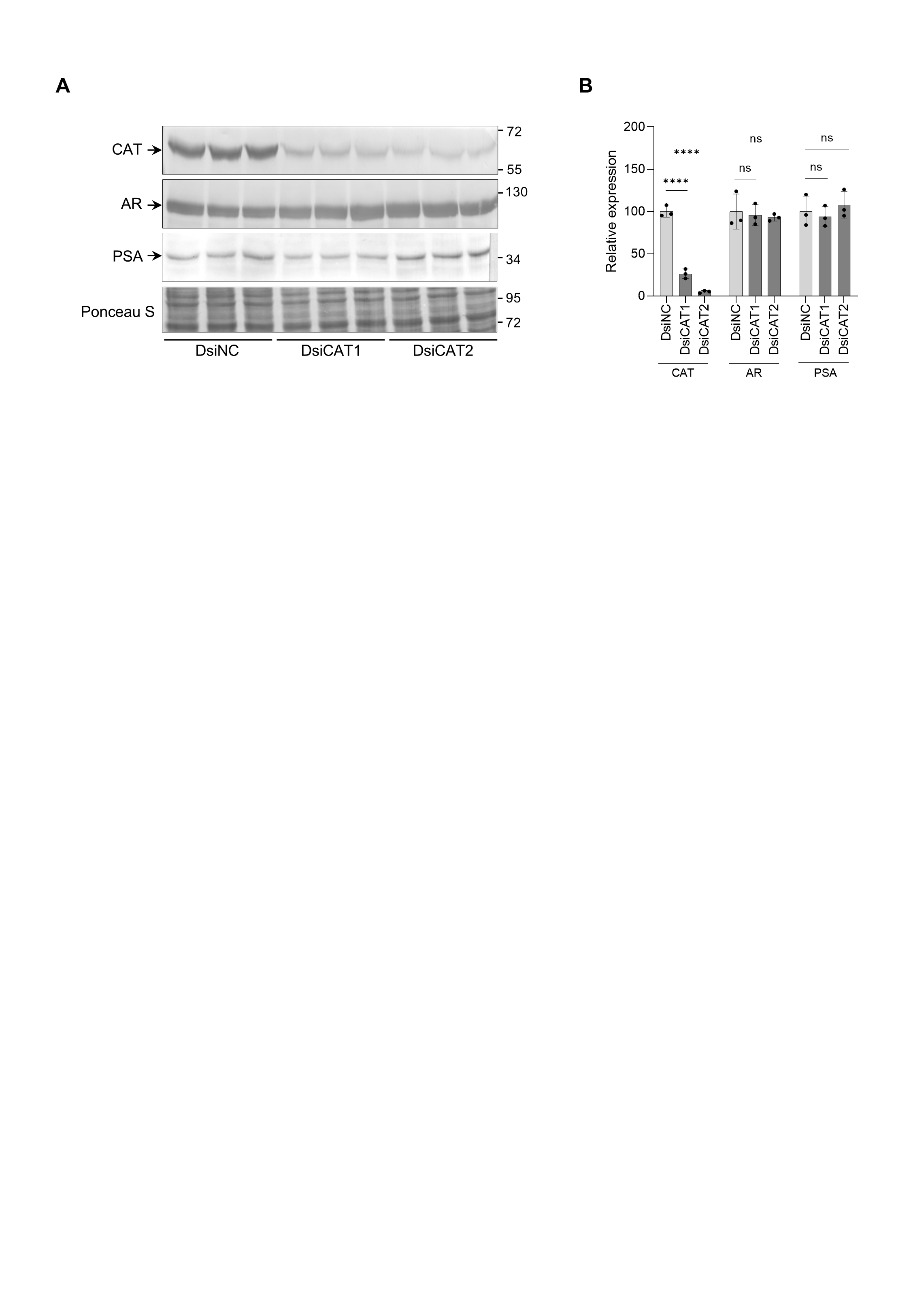

Supplement: Supplementary file 1 [file antioxidants-13-01340-s001.zip › Hussein et al_Figure S11.png]

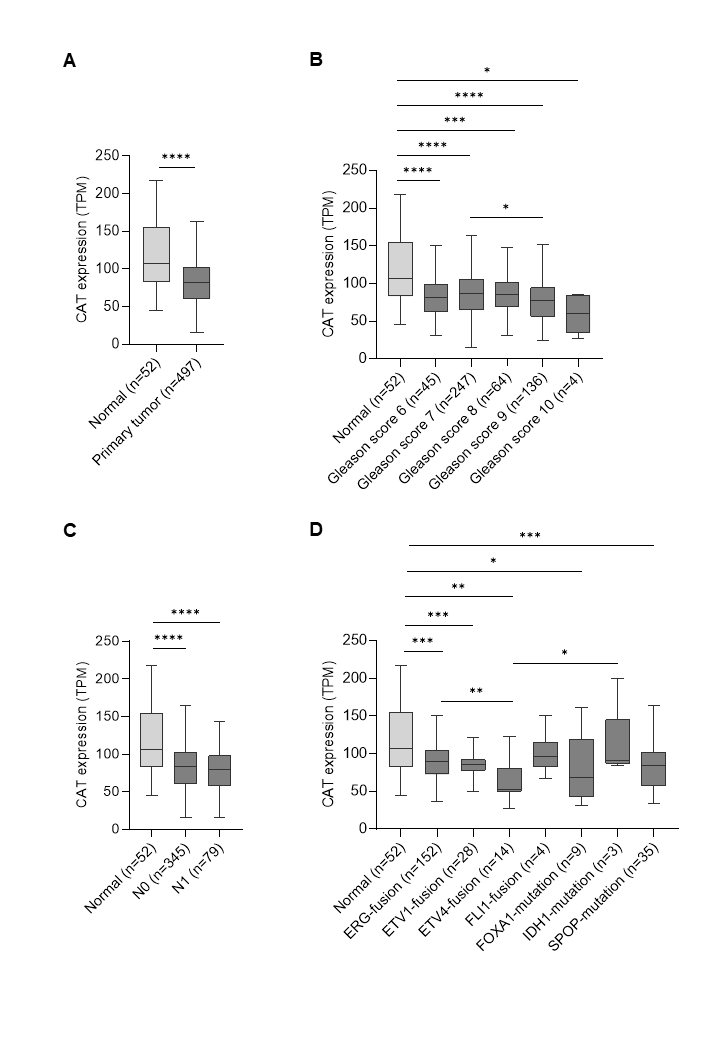

Supplement: Supplementary file 1 [file antioxidants-13-01340-s001.zip › Hussein et al_Figure S12.png]

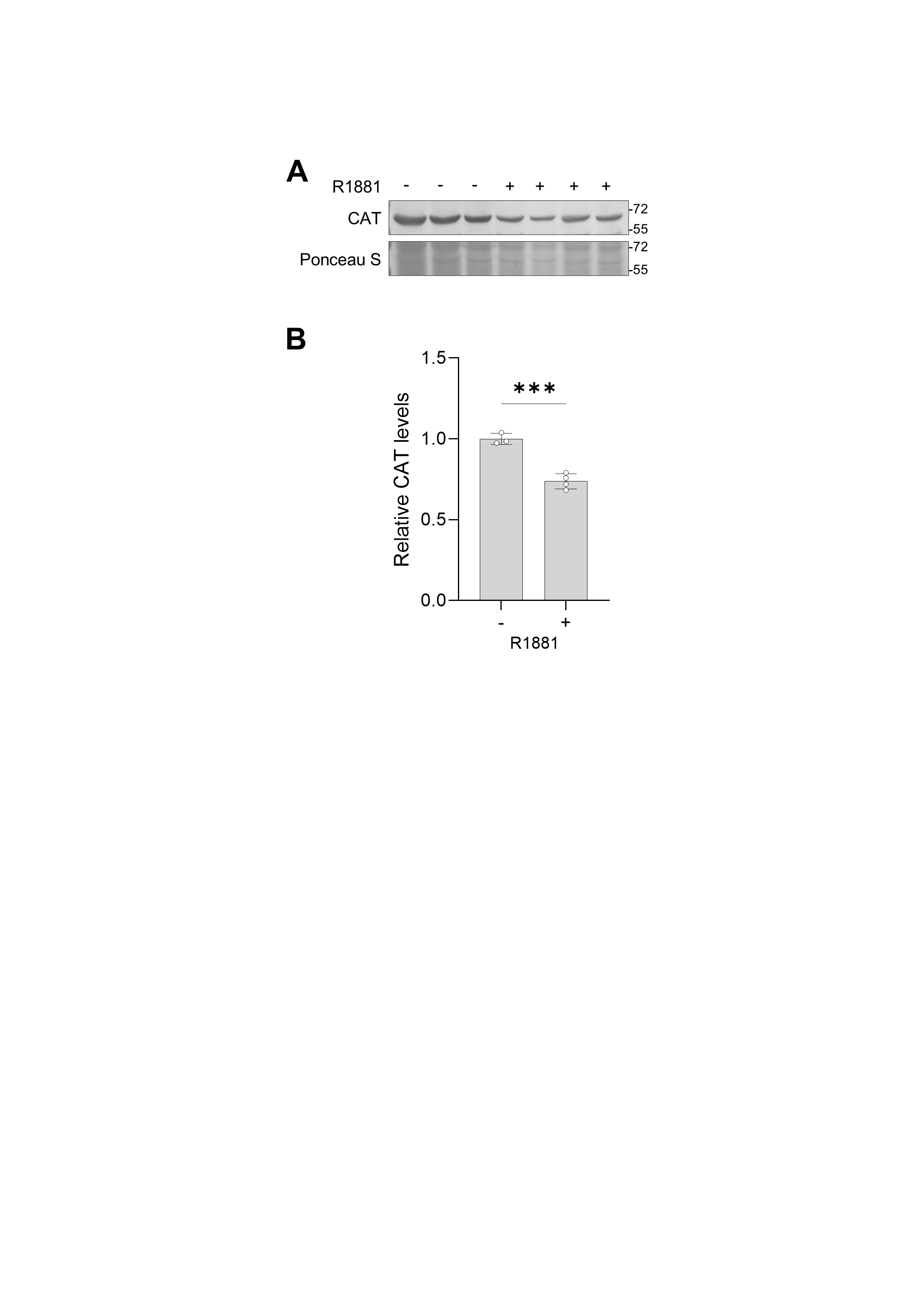

Supplement: Supplementary file 1 [file antioxidants-13-01340-s001.zip › Hussein et al_Figure S13.png]

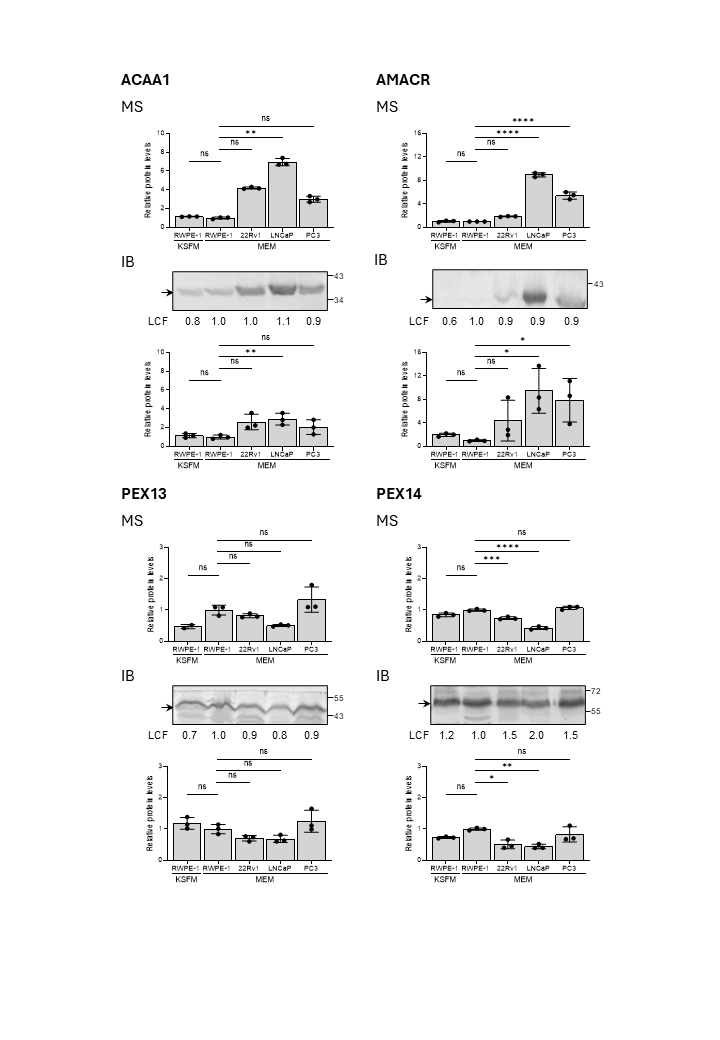

Supplement: Supplementary file 1 [file antioxidants-13-01340-s001.zip › Hussein et al_Figure S2.png]

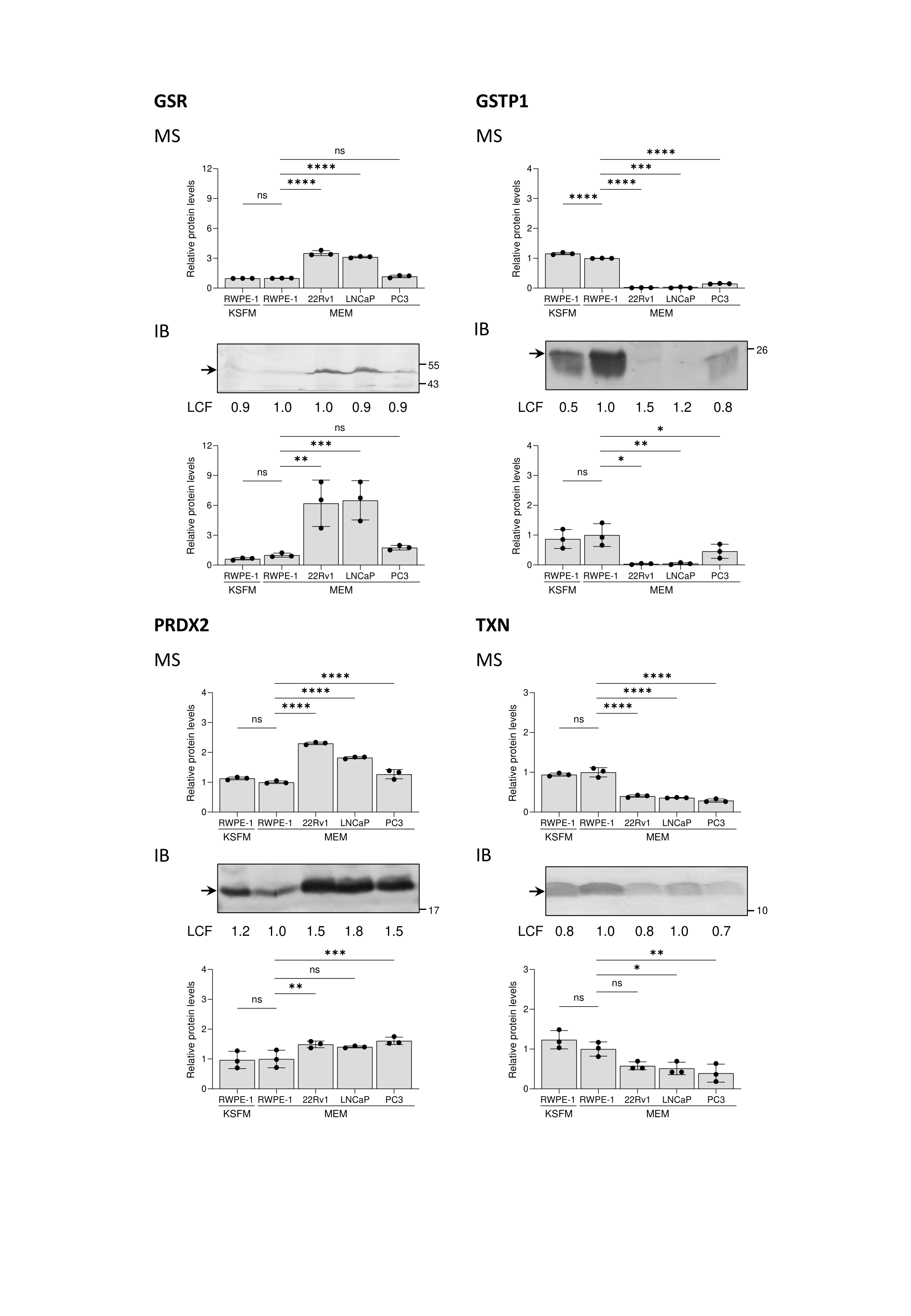

Supplement: Supplementary file 1 [file antioxidants-13-01340-s001.zip › Hussein et al_Figure S3.png]

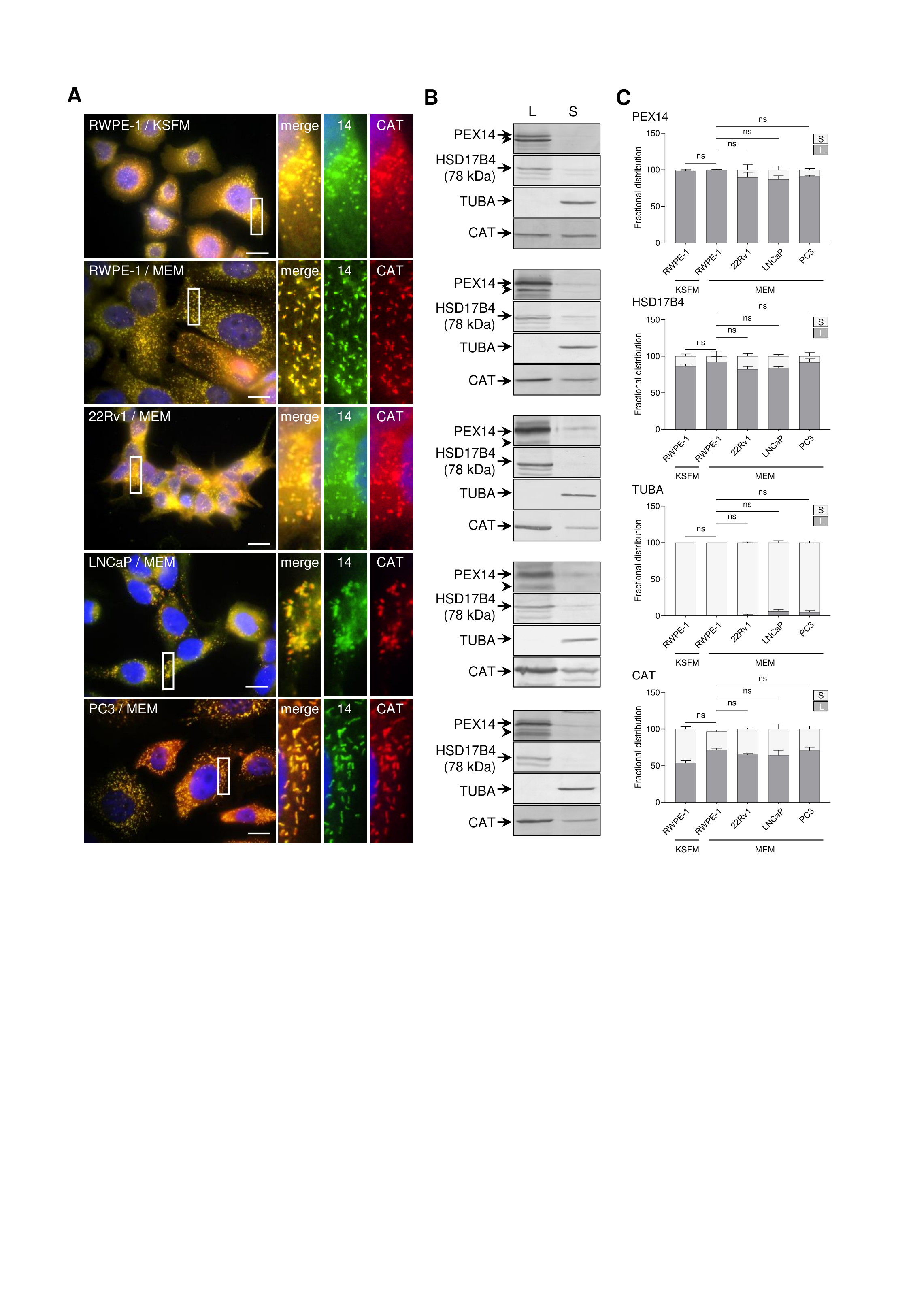

Supplement: Supplementary file 1 [file antioxidants-13-01340-s001.zip › Hussein et al_Figure S4.png]

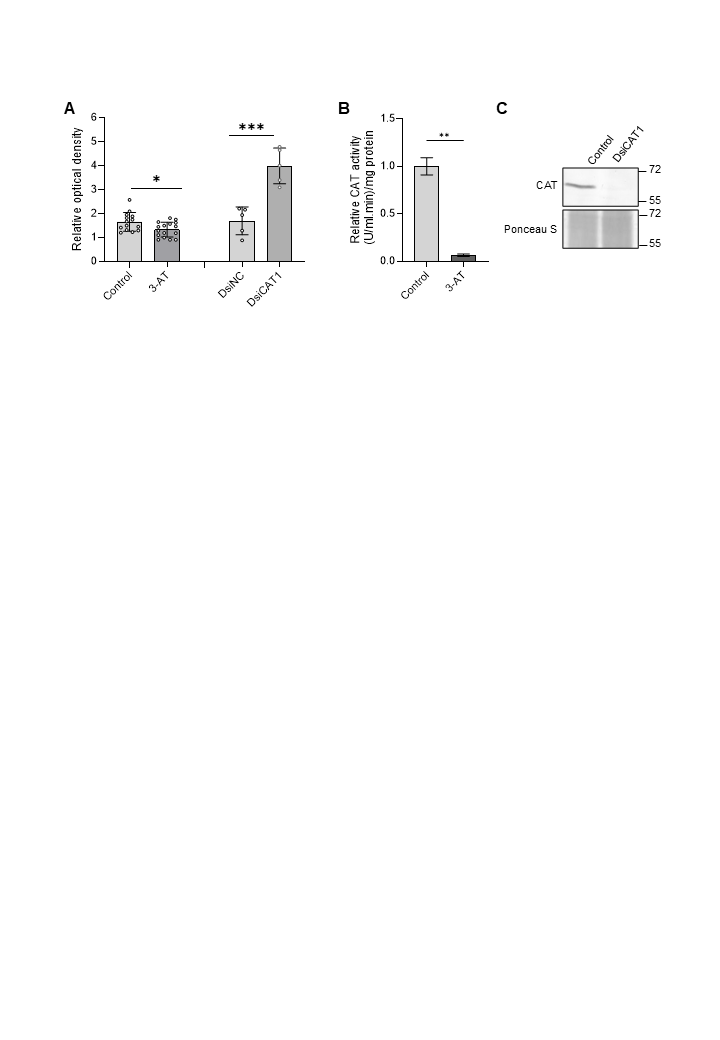

Supplement: Supplementary file 1 [file antioxidants-13-01340-s001.zip › Hussein et al_Figure S5.png]

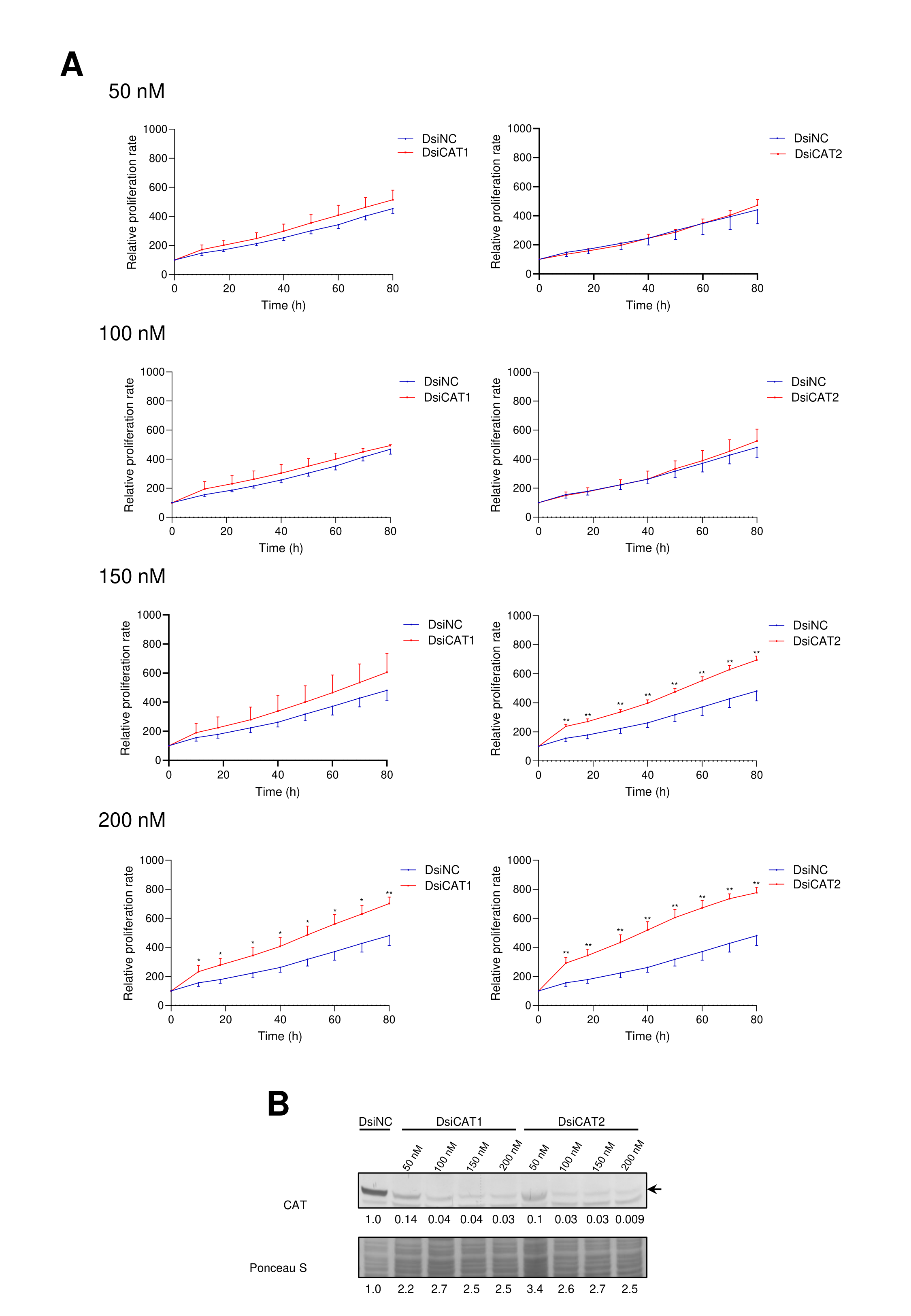

Supplement: Supplementary file 1 [file antioxidants-13-01340-s001.zip › Hussein et al_Figure S6.png]

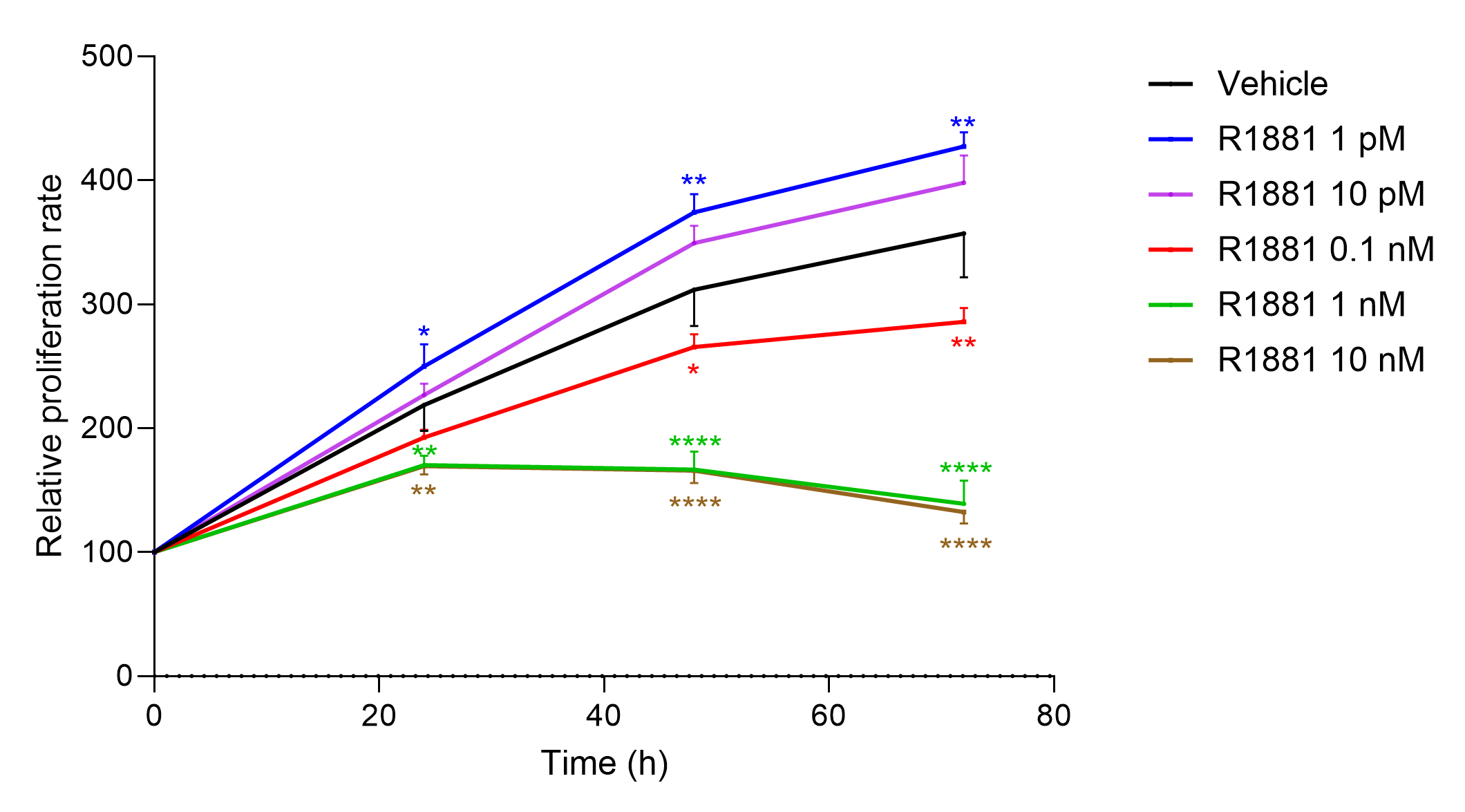

Supplement: Supplementary file 1 [file antioxidants-13-01340-s001.zip › Hussein et al_Figure S7.png]

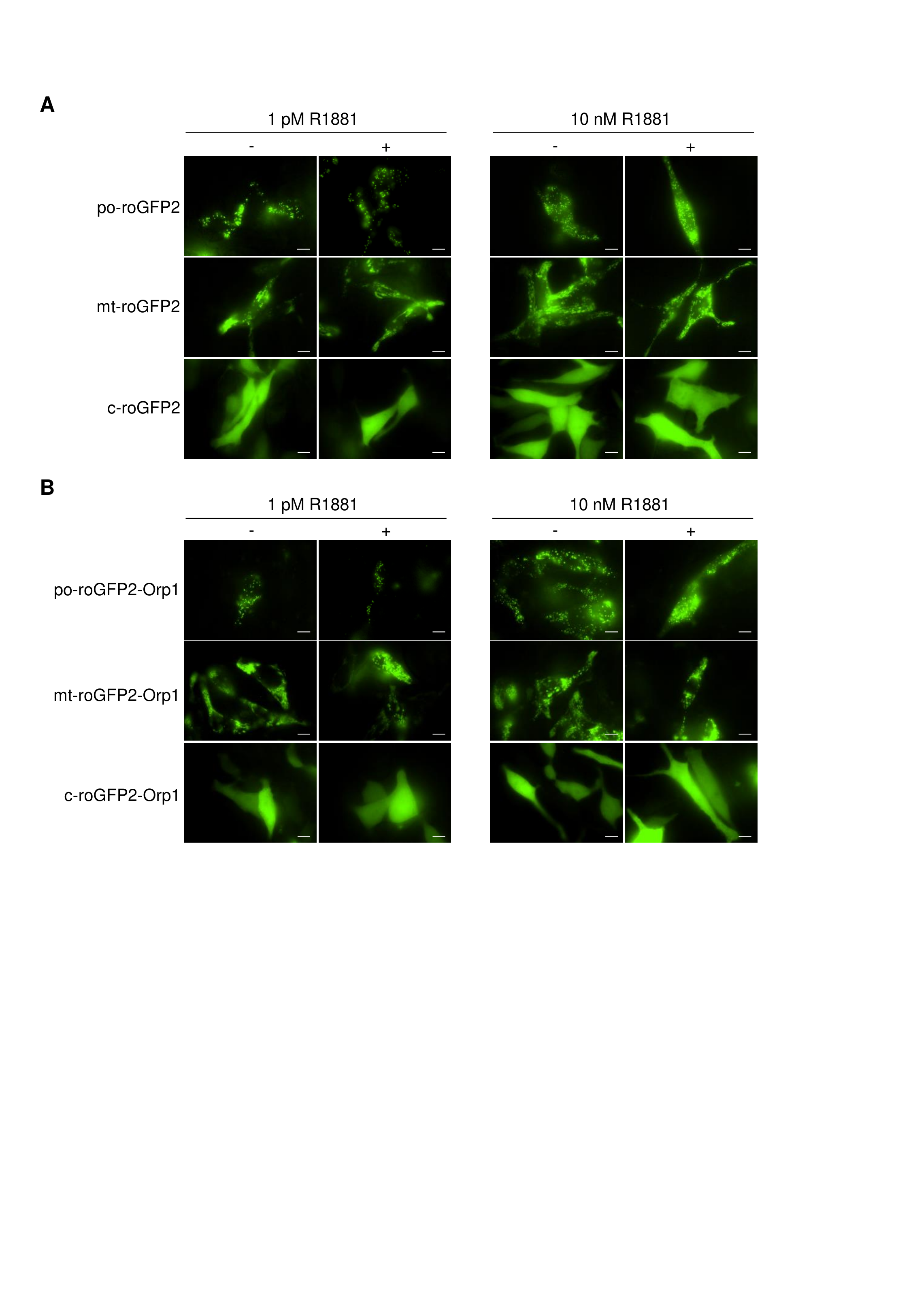

Supplement: Supplementary file 1 [file antioxidants-13-01340-s001.zip › Hussein et al_Figure S8.png]

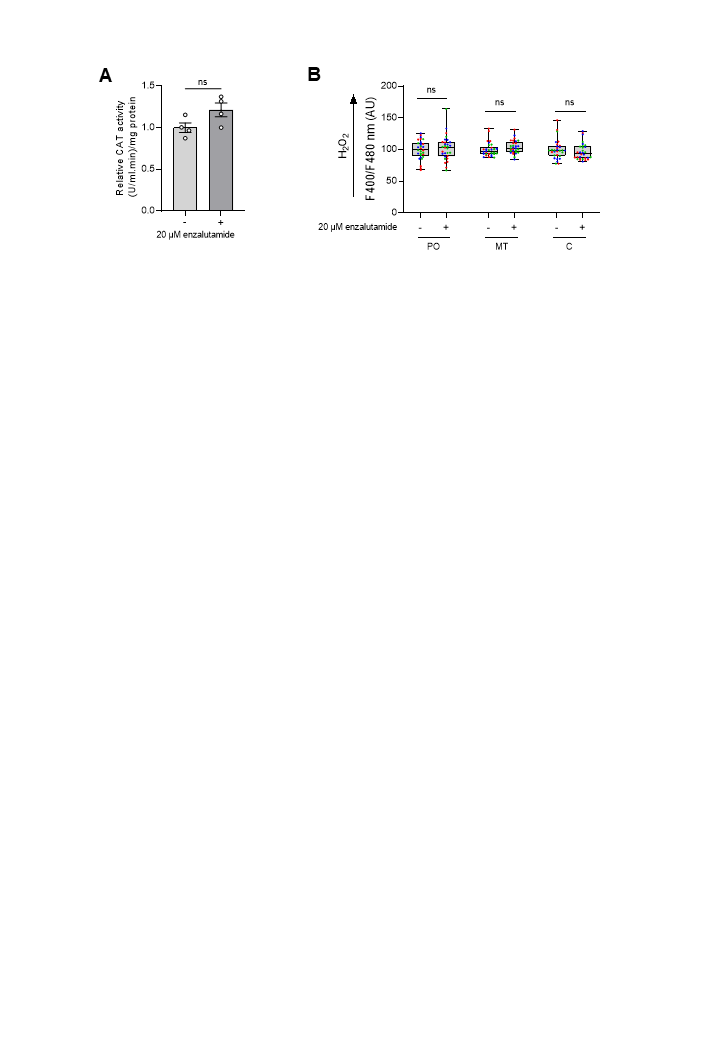

Supplement: Supplementary file 1 [file antioxidants-13-01340-s001.zip › Hussein et al_Figure S9.png]
